# The water chemistry and microbiome of household wells in Medawachchiya, Sri Lanka, an area with high prevalence of chronic kidney disease of unknown origin (CKDu)

**DOI:** 10.1038/s41598-020-75336-7

**Published:** 2020-10-26

**Authors:** Liza K. McDonough, Karina T. Meredith, Chandima Nikagolla, Ryan J. Middleton, Jian K. Tan, Asanga V. Ranasinghe, Frederic Sierro, Richard B. Banati

**Affiliations:** 1 Australian Nuclear Science and Technology Organisation (ANSTO), New Illawarra Rd, Lucas Heights, NSW 2234 Australia; 2 School of Biological, Earth and Environmental Sciences, UNSW Sydney, Sydney, NSW 2052 Australia; 3 School of Civil and Environmental Engineering, Science and Engineering Faculty, Queensland University of Technology, Brisbane, QLD 4000 Australia; 4 Charles Perkins Centre, University of Sydney, Sydney, NSW Australia; 5 Faculty of Medicine and Health, University of Sydney, Sydney, NSW Australia; 6 National Renal Disease Prevention and Research Unit, Ministry of Health, Colombo 10, Sri Lanka

**Keywords:** Water microbiology, Environmental chemistry, Chronic kidney disease

## Abstract

Chronic kidney disease (CKD) of unknown etiology (CKDu) mostly affects agricultural communities in Central America, South Asia, Africa, but likely also in North America and Australia. One such area with increased CKDu prevalence is the Medawachchiya District Secretariat Division of the Anuradhapura District in the North Central Province of Sri Lanka. Recent research has focused on the presence of various microbial pathogens in drinking water as potential causal or contributing factors to CKDu, yet no study to date has performed a more comprehensive microbial and water chemistry assessment of household wells used for domestic water supply in areas of high CKDu prevalence. In this study, we describe the chemical composition and total microbial content in 30 domestic household wells in the Medawachchiya District Secretariat Division. While the chemical composition in the tested wells mostly lies within standard drinking water limits, except for high levels of fluoride (F), magnesium (Mg), sodium (Na), chloride (Cl) and calcium (Ca) in some samples, we find a frequent presence of cyanotoxin-producing *Microcystis*, confirming earlier studies in Sri Lanka. Since the total microbial content of drinking water also directly influences the composition of the human gut microbiome, it can be considered an important determinant of health. Several bacterial phyla were previously reported in the gut microbiome of patients with CKD. Using these bacteria phyla to define operational taxonomic units, we found that these bacteria also occur in the microbiome of the sampled well water. Based on available environmental data, our study demonstrates associations between the abundances of these bacteria with geographical distribution, well water temperature and likely fertilizer use in the local surface water catchment area of the individual household wells. Our results reinforce the recommendation that household wells with stagnant or infrequently used water should be purged prior to use for drinking water, bathing and irrigation. The latter is suggested because of the reported potential accumulation of bacterial toxins by agricultural crops. The observation that bacteria previously found in chronic kidney disease patients are also present in household wells requires a more detailed systematic study of both the human gut and drinking water microbiomes in CKDu patients, in relation to disease prevalence and progression.

## Introduction

The 2016 Annual Health Statistics of Sri Lanka lists chronic kidney disease (CKD) as the leading cause of hospital deaths in Anuradhapura and Polonnaruwa, the two districts of North Central Province^1^. The above average prevalence of kidney disease is attributed to a chronic kidney disease of unknown or undetermined origin (CKDu), that appears to have emerged over the past 20 years. Though there is neither full consensus nor a comprehensive case definition, by general agreement the disease is not associated with known risk factors for CKD, such as diabetes or hypertension, and occurs mostly in poorer young and middle-aged individuals in agricultural communities. Progressive and asymptomatic until the late stages, its characteristic chronic tubulointerstitial tissue pathology with secondary glomerulosclerosis^2^ has recently resulted in a change of terminology from CKDu to CINAC, chronic interstitial nephritis in agricultural communities (CINAC)^3^.

In Sri Lanka’s North Central region, notably the Medawachchiya District Secretariat Division of the Anuradhapura District which has some of the highest prevalence of CKDu^4^, 80% of the mostly farming population relies on groundwater for their daily water needs^5^, including drinking water. Previous reports have investigated potential environmental^6,7^, genetic^8^, occupational and social^3,9,10^ risk factors for CKDu without generating conclusive evidence for specific causes. Recent studies have pointed towards an association between CKDu, groundwater chemistry and the water quality in household wells^11–14^, suggesting that the disease could be caused by hydrogeochemical factors such as high fluoride and water hardness, heavy metals and microbial contamination or combinations thereof. CKDu is presently viewed as a complex disease mostly found in certain rural populations and provisionally defined by the absence of consistently observed causes commonly associated with chronic kidney disease. Recent research supports the hypothesis that CKDu is likely caused by a combination of intrinsic and external factors such as malnutrition, dehydration, and the consumption of poor quality drinking water including those high in fluoride (F) concentrations^14,15^, or pathogens and bacterial toxins. However, the actual hierarchy of importance of the various contributing disease causes or, importantly, any risk-enhancing or mitigating interactions between these remain to be established^15–18^. Heavy metal exposure from groundwater has largely been ruled out as the major causal factor for CKDu in Sri Lanka^7,19^, however, contamination by pathogens and bacterial toxins in water used for irrigation, domestic and potable water supply remains a valid hypothesis^17,18,20–23^.

In particular, the toxin-producing cyanobacteria genera, including *Microcystis* and *Cylindrospermopsis*, as well as *Leptospira interrogans* can lead to kidney disease^14,18,24,25^ and have, therefore, been put forward as potential causes of CKDu^21,26,27^. Significant increases of cyanobacteria in freshwater resources in Sri Lanka have occurred over the past century as a result of eutrophication and rising temperatures^21,28^, demonstrating that altered water chemistry is likely to promote the presence of pathogenic bacteria in this region.

There has not yet been a study that specifically probes into the association of the water chemistry in individual household wells with the composition of their complete microbiome.

We suggest that well water chemistry, including temperature, pH, dissolved oxygen (DO) and nutrients (phosphorus (P),potassium (K) and nitrate (NO_3_)) will favor the emergence of typical well water microbiomes, that are both determined by and determining the chemical characteristics of the well water.

The sampling for this project was undertaken, in an area of Sri Lanka which is highly affected by CKDu, as a pilot study to determine possible links between altered water chemistry and the microbiome of household well water used as a regular or occasional drinking, washing or cooking water source. The rationale for the total microbiome analysis of household well water beyond those bacteria already known to produce nephrotoxins is based on the most recent observation that the source and microbial content of drinking water (in addition to food) itself significantly determines the microbial composition of the human gut microbiome^29–31^. In turn, it is increasingly recognized that an individual’s human microbiome composition has a close, disease modifying, association across a wide range of health and disease conditions, including CKD^32^.

## Methods

### Sampling

**Figure 1:**
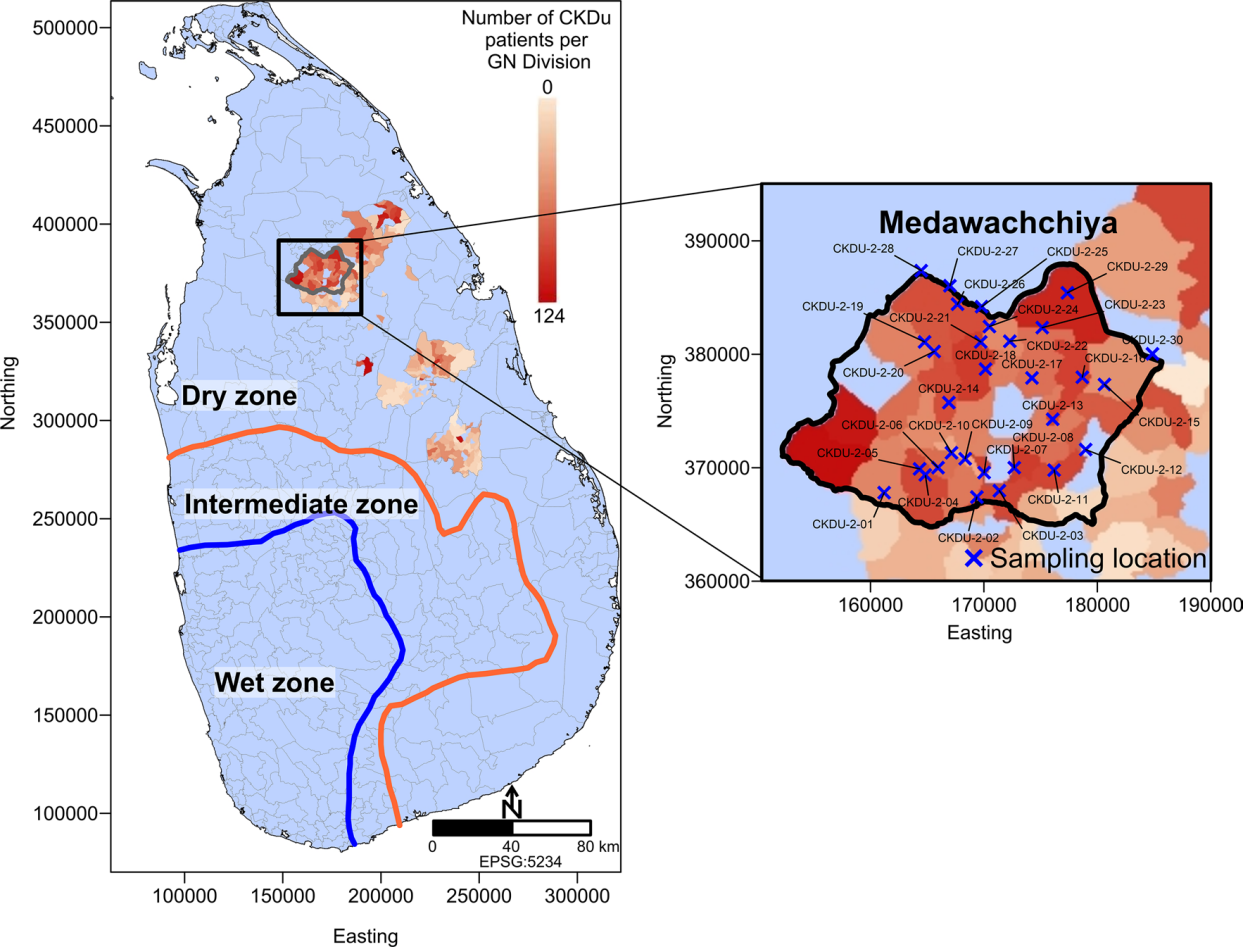
Sri Lanka map showing dry (< 1750 mm year^−1^), intermediate (1750–2500 mm year^−1^) and wet (> 2500 mm year^−1^) zones. Inset shows the study region in Medawachchiya with groundwater sampling locations shown as blue crosses. CKDu prevalence represents numbers of CKDu patients in each Grama Niladhari (GN) Division, showing high prevalence within the dry zone. Figure prepared in Surfer v.11.0.642 (www.goldensoftware.com/products/surfer).

Thirty domestic well water samples, comprised of shallow groundwater (between 1 and 9 m depth below ground surface), were collected from the Medawachchiya region between the 15th of April 2018 and 23rd of April 2018. Sample locations are shown in Fig. 1.

Sampled water was obtained from a combination of lined and unlined wells and springs. During sampling, lining materials were observed to be either brick, concrete, or a combination of both. Rock types in the wells were predominantly weathered quartz-feldspathic gneiss overlain with between 0–9 m of soil, surrounded by coconut trees, rice paddies, grass, bushland and home gardens.

To sample, a peristaltic pump was placed 1 m (m) below the standing water level where possible, or between the base of the well and the surface where standing water level was < 1 m. Electrical conductivity (EC), water temperature, dissolved oxygen (DO) and pH were measured using a flow cell connected to the pump and a YSI meter. During pumping wells simultaneously refilled with water, therefore water was pumped until field parameters stabilized prior to sampling. A high flow Waterra 0.45 µm filter was then connected to the pump outlet. Each sample bottle was rinsed three times with the filtered water sample and then filled. Cation samples were collected in 60 ml high-density polyethylene (HDPE) containers respectively, acidified using 0.4 ml of ultrapure nitric acid (HNO_3_) and refrigerated. Anion samples were collected in 60 ml HDPE bottles. Stable water isotope samples were collected in 2 × 30 ml HDPE bottles. Samples collected for the measurement of ^13^C of dissolved inorganic carbon (^13^C_DIC_) were collected in 2 × 12 ml glass vials ensuring no headspace. The samples were inverted to ensure no bubbles were present and refrigerated after collection. The filter unit was retained for microbiome analyses and frozen. The particulate organic carbon (^13^C_POC_) samples were collected by adding a Millipore POC filter with 0.7 µm filter paper to the peristaltic pump outlet and ~ 2 L of water were passed through the filter. ^13^C_POC_ samples were then frozen. Tape was placed around the lid of all water samples to limit atmospheric exchange.

Samples were removed from the refrigerator or freezer when field work was complete and the samples were ready to be posted. Frozen samples were packed with other frozen samples in a foam cool box to ensure they would stay as cold as possible during transport. Once samples arrived at Australian Nuclear Science and Technology Organisation (ANSTO), samples for freezing or refrigeration were immediately flash frozen and refrigerated respectively and gamma-irradiated at 50 kGray prior to transfer to the respective labs for analysis at ANSTO.

### Analysis

All water samples were analysed at Australian Nuclear Science and Technology Organisation (ANSTO). Major element compositions and trace element compositions were determined by inductively coupled plasma atomic emission spectroscopy (ICP-AES) using a Varian 820MS and inductively coupled plasma mass spectrometry (ICP-MS) using a Thermo Fisher iCAP7600 Duo respectively. Anions were measured by ion chromatography using a Dionex ICS-2100. The number of decimal places in the lab reports are determined for each sample based on the relative standard deviation (RSD) of the lab standard for the element at the relevant sample concentration. For ICPAES, ICPMS and IC, RSD are between 1 and 5% for data that are 10 times the limit of reporting (LOR). For samples with data that are < 10 times the LOR, the RSD are > 5%, and up to 50% if the sample is at the LOR. Stable water isotopes and stable dissolved inorganic carbon (DIC) isotopes were measured using Gas Bench II coupled to a continuous flow delta V advantage isotope ratio mass spectrometer. A two-point calibration was used to normalize the data which consisted of two standards that bracket the samples being analyzed. The results were reported relative to IAEA secondary standards which are certified relative to VPDB using a two point calibration, with final sample results accurate to ± 0.3 ‰ for δ^13^C_DIC_. The POC isotopes were measured using an elemental analyzer-isotopic ratio mass spectrometer.

To perform the microbiome analysis, DNA was extracted from the filters using the PowerSoil DNA isolation kit (MO BIO Laboratories) following the manufacturer’s instructions. The variable V3 and V4 regions of the 16S rRNA gene was then amplified via PCR (16S Forward primer: 5′-TCGTCGGCAGCGTCAGATGTGTATAAGAGACAGCCTACGGGNGGCWGCAG and 16S Reverse primer: 5′-GTCTCGTGGGCTCGGAGATGTGTATAAGAGACAGGACTACHVGGGTATCTAATCC). Each reaction consisted of 1 × KAPA HiFi HotSart ReadyMix (KAPA Biosystems), 0.2 µM of each primer and 12.5 ng of sample DNA in a final volume of 25 µl. The thermocycler conditions were 95 °C for 3 min, then 25 cycles at 98 °C for 30 s, 55 °C for 30 s and 72 °C for 30 s, then a final extension at 72 °C for 5 min. PCR products were then purified using AMPure XP beads (Beckman Coulter) following the Illumina 16S Metagenomic Sequencing Library preparation guide. At this point, samples were transferred to the Ramaciotti Centre for Genomics (UNSW Sydney, Australia) for further processing. Briefly, dual indices and Illumina sequencing adaptors were attached using the Nextera XT Index Kit. Samples were cleaned again using AMPure XP beads and then quantified, normalized and pooled before being sequenced on the Illumina MiSeq platform using paired 300 bp reads and MiSeq v3 reagents.

QIIME (Quantitative Insights Into Microbial Ecology) version 1.9.1 was used to process the sequence data^33^. The mapping file was validated using validate_mapping_file.py. Forward and reverse reads were then joined using the script multiple_join_paired_ends.py and then multiple_extract_barcodes.py was used to remove the primer sequences and process the data for subsequent steps. Further processing and quality filtering was performed using multiple_split_libraries_fastq.py, chimeras were identified with identify_chimeric_seqs.py and the usearch tool, then the chimeras removed with filter_fasta.py. Finally, operational taxonomic units (OTUs) were picked using pick_open_reference_otus.py, usearch and the SILVA rRNA database release 132. OTUs with total counts across all samples of less than 10 were removed.

Statistical analyses and figures were generated in R (v3.5.1). Species accumulation curves were generated using the vegan package. Stacked barplots, heatmaps and microbial networks were generated using the physeq package. Redundancy analysis (RDA) and principle component analysis (PCA) were performed in R and used to compare microbes to water chemistry parameters, and group co-occurrent CKD-associated microbe groups respectively. Count data have been used as scaling problems were encountered due to some microbes being present in very low abundances. Correlations between microbes and water chemistry parameters were also performed in R. Quantile–Quantile plots and constant variance were checked for linear correlations to ensure normality and constant variance of residuals. Where data was heavily skewed, the response variable was log or square root transformed to normalize. Non-parametric Spearman correlations were used where data remained heavily skewed after transformation.

## Results

### Geochemistry

Piper plots (Supplementary Figure 1) show that the sampled water types include calcium chloride and mixed water types^34^. Table 1 summarizes the concentration of various inorganic water quality parameters measured in this study compared to the World Health Organization (WHO) and Sri Lanka Standards Institution (SLSI) guideline values for drinking water^35,36^. Our data show high levels of fluoride (F) in the Medawachchiya region with 30% of wells sampled exceeding the WHO guideline value of 1.5 mg L^−1^ for F, and 57% exceeding the Sri Lanka specification of 1.0 mg L^−1^ for F in potable water. All other parameters including chromium (Cr), arsenic (As), barium (Ba), uranium (U), copper (Cu), nitrate (NO_3_) and nickel (Ni) fall below the WHO guideline values for drinking water for all samples (Table 1).

**Table 1:** World Health Organization (WHO) and Sri Lanka drinking water standard 614:2013 guideline values for inorganic water chemistry parameter limits in drinking water compared against values observed in this study.

Parameter	WHO health-based limit^35^	This study (n = 30)					
		Sri Lanka standard 614 : 2013^36^ limit	Min	Median	Average	Max	Standard deviation
As (mg L^−1^)	0.010	0.010	0.005	0.005	0.005	0.005	< 0.005
Ba (mg L^−1^)	0.700	–	0.033	0.200	0.234	0.597	0.156
Ca (mg L^−1^)	–	100.0	8.3	71.7	74.1	140.0^a^	30.3
Cl (mg L^−1^)	–	250.0	17.7	65.4	97.5	310.0^b^	80.8
Cr (mg L^−1^)	0.050	0.050	0.001	0.001	0.001	0.002	< 0.001
Cu (mg L^−1^)	2.000	1.000	0.001	0.001	0.001	0.002	< 0.001
F (mg L^−1^)	1.50	1.00	0.07	1.11	1.24	3.70^c^	0.74
K (mg L^−1^)	–	–	0.6	1.4	3.5	54.0	9.6
Mg (mg L^−1^)	–	30.0	5.6	33.9	39.7	95.2^d^	19.9
Na (mg L^−1^)	–	200.0	10.9	33.9	82.3	234.0^e^	19.9
Ni (mg L^−1^)	0.070	0.020	0.001	0.002	0.003	0.008	0.002
NO_3_ (mg L^−1^)	50.0	50.0	0.5	0.5	1.9	12.4	3.1
P (mg L^−1^)	–	–	0.01	0.01	0.05	0.24	0.07
U (mg L^−1^)	0.030	–	0.001	0.001	0.001	0.006	0.001
Zn (mg L^−1^)	–	3.000	0.001	0.002	0.003	0.008	0.002

For the purposes of this assessment, samples falling below the limit of detection have been assigned a value equal to the limit of detection.

^a^4 of 30 samples exceed the SLSI guideline value.

^b^3 of 30 samples exceed the SLSI guideline value.

^c^17 of 30 samples exceed the SLSI guideline value, 9 of 30 samples exceed the WHO guideline value.

^c^21 of 30 samples exceed the SLSI guideline value.

^d^1 of 30 samples exceed the SLSI guideline value.

### Microbial diversity

**Figure 2:**
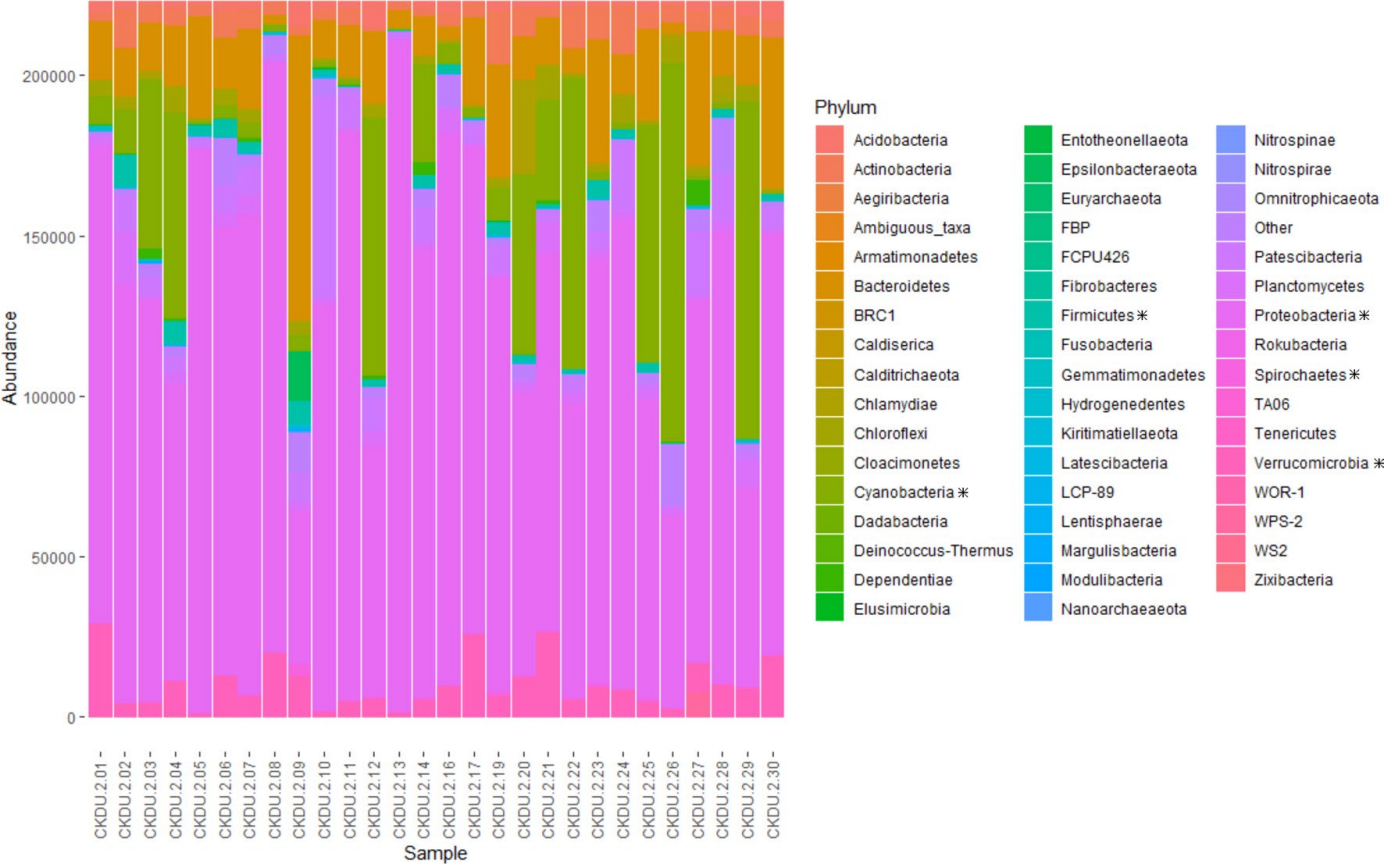
Stacked bar chart showing abundance (counts) of microbial phyla in each sample. Data normalized to the median sequencing depth. NB: microbe data for samples CKDU-2-15 and CKDU-2-18 not available. Phylum shown with an asterisk symbol represent groups containing microbes identified in higher concentrations in CKD patients compared to healthy patients. These include Verrucomicrobia, Gammaproteobacteria, Enterobacteriacea, Methylobacterium and Desulfovibrio (of the Proteobacteria phylum), Leptospira (of the Spirochaetes phylum), and Holdemania, Turicibacter and Clostridium sensu stricto (of the Firmicutes phylum).

A total of 58 unique phyla categories were detected (Fig. 2). The microbial composition is dominated by 16 phyla which have abundances of ≥ 1%. These include Acidobacteria, Actinobacteria, Bacteroidetes, Chlamydiae, Chloroflexi, Cyanobacteria, Dependentiae, Epsilonbacteraeota, Firmicutes, Unassigned bacteria (Other), Patescibacteria, Planctomycetes, Proteobacteria, Spirochaetes, Verrucomicrobia and WPS-2 (Fig. 2).

**Figure 3:**
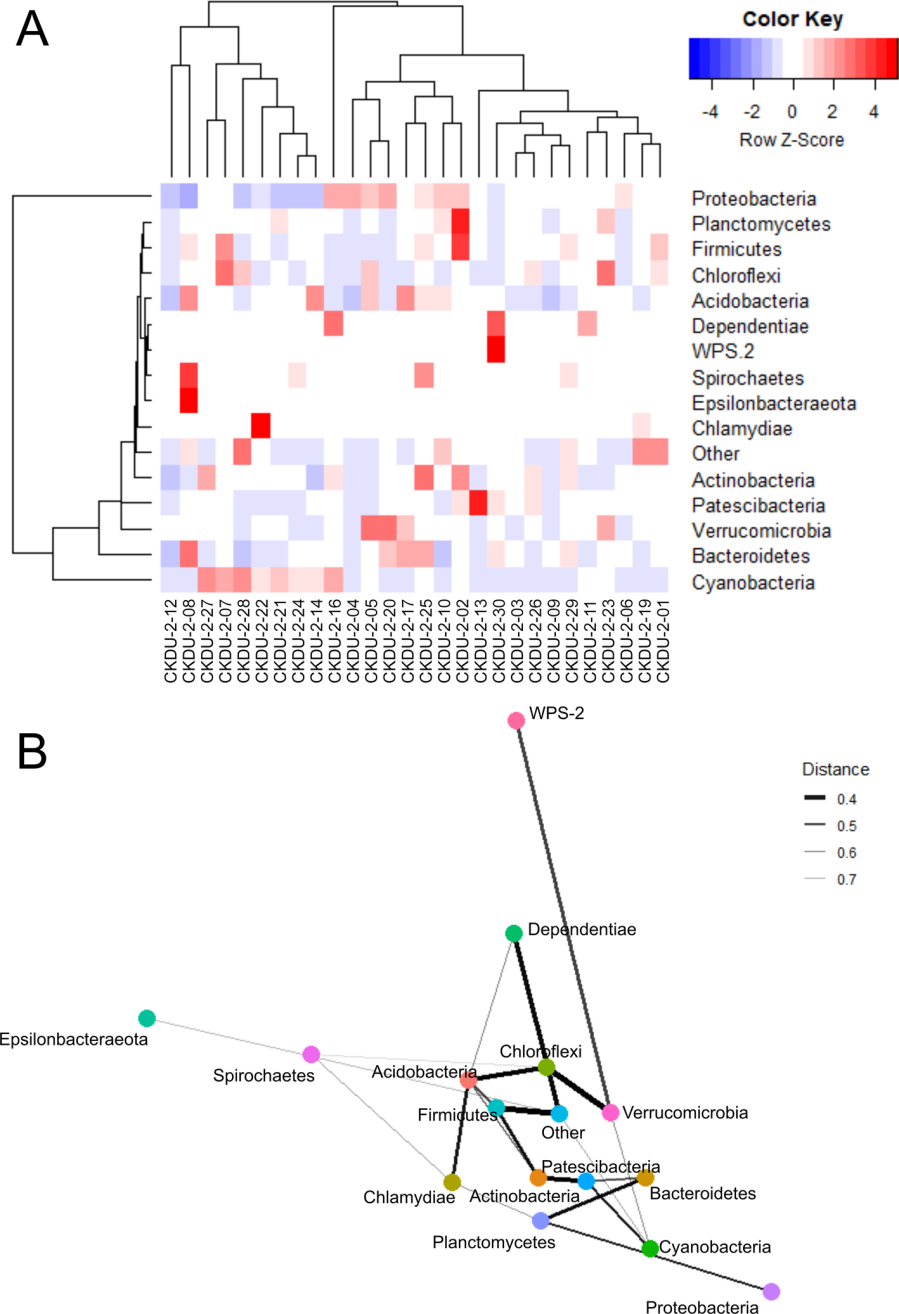
(**A**) Heatmap of microbial phyla (> 1% abundance), with cluster analyses for sample grouping (upper cluster diagram) and microbial groupings (left cluster diagram). Data are scaled by rows to have a mean of zero and a standard deviation of one. (**B**) Network map based on Bray–Curtis distance showing similarities between microbial genera counts for phyla with abundance > 1%. Thicker lines represent greater similarity between phyla counts, thinner lines represent less similarity between phyla counts. Data normalized to the median sequencing depth.

A heatmap of microbial counts in each sample and co-occurrence networks for these more abundant phyla show a negative association between Proteobacteria and Cyanobacteria for most samples (Fig. 3). Sample CKDU-2-04 however shows high Proteobacteria and Cyanobacteria counts, whilst CKDU-2-08, CKDU-2-12 and CKDU-2-30 show low Proteobacteria and low Cyanobacteria. Figure 3B indicates an association between Cyanobacteria, Patescibacteria and Verrucomicrobia.

Species accumulation curves (Supplementary Figure 2) indicate that there is a relatively low proportion of diversity in the samples, with 80% of phyla identified in the total dataset reached within approximately 4 of the 28 samples, and 95% reached within approximately 15 of the 28 samples. This suggests that sampling an additional 13 sites would only result in the identification of approximately 3 additional phyla which may not have been present at the other 15 sampling sites. 80% of the genera are reached within approximately 7 of the samples, and 95% reached within approximately 19 of the 28 samples, suggesting that the sampling of an additional 9 sites resulted in the identification of approximately 83 additional microbial genera.

#### Presence and geospatial mapping of previously identified CKD-associated bacteria

We identify bacteria which have been positively associated with CKD in previous studies^37–39^. These include *Verrucomicrobia*, *Gammaproteobacteria*, *Enterobacteraceae*, *Microcystis*, *Leptospira*, *Clostridium *sensu stricto, *Desulfovibrio*, *Holdemania*, *Turicibacter* and *Methylobacterium* (Table 2), which have been identified in higher abundances in CKD fecal samples compared to healthy patients^37–39^. The spatial distributions of these microbes are shown in Supplementary Figure 3.

**Table 2:** Total abundance of bacteria for all samples shown as a percentage of the total microbial count in all samples, operational taxonomic unit (OTU) level and sample average, minimum, maximum and standard deviations values of microbial counts of microbes in water samples from this study.

	OTU level	Total abundance for all water samples (%)	Counts			
			Average	Minimum	Maximum	σ
Verrucomicrobia	Phylum	4.82	10,727	913	41,624	10,378
Gammaproteobacteria	Class	32.48	124,678	37,282	237,360	56,297
Enterobacteriaceae	Family	0.01	30	0	229	49
Microcystis	Genus	0.40	885	0	22,187	4193
Leptospira	Genus	0.01	21	0	217	48
Clostridium sensu stricto	Genus	0.24	542	18	4482	1097
Desulfovibrio	Genus	0.03	73	0	1243	239
Holdemania	Genus	3.05 × 10^−4^	1	0	12	2
Turicibacter	Genus	0.05	103	0	2272	429
Methylobacterium	Genus	3.12	6936	237	36,431	7878

Table shows the current study’s results for microbes previously identified^37–39^ in higher concentrations in CKD compared to healthy individuals.

However, we do not identify any *Clostridium IV*, *Enterococcus* or *Paraprevotella* or only identify very small counts of Alloprevotella (up to 2 counts in 4 wells, which may be a result of sequencing error) all of which have also been shown to be enriched in CKD patient stool^37^. We did not identify any cyanotoxin-producing *Cylindrospermopsis*, but do observe the cyanotoxin-producing genus *Microcystis*, both of which can cause kidney damage^14,18,25^. We identify only a very small number of *Akkermansia* (11 counts), found only in one well (CKDU-2-04). *Akkermansia* is a probiotic which has been shown to be significantly higher in abundance in healthy individuals compared to those with CKD^37^.

#### Water chemistry impacts on CKD-related bacteria counts

**Figure 4:**
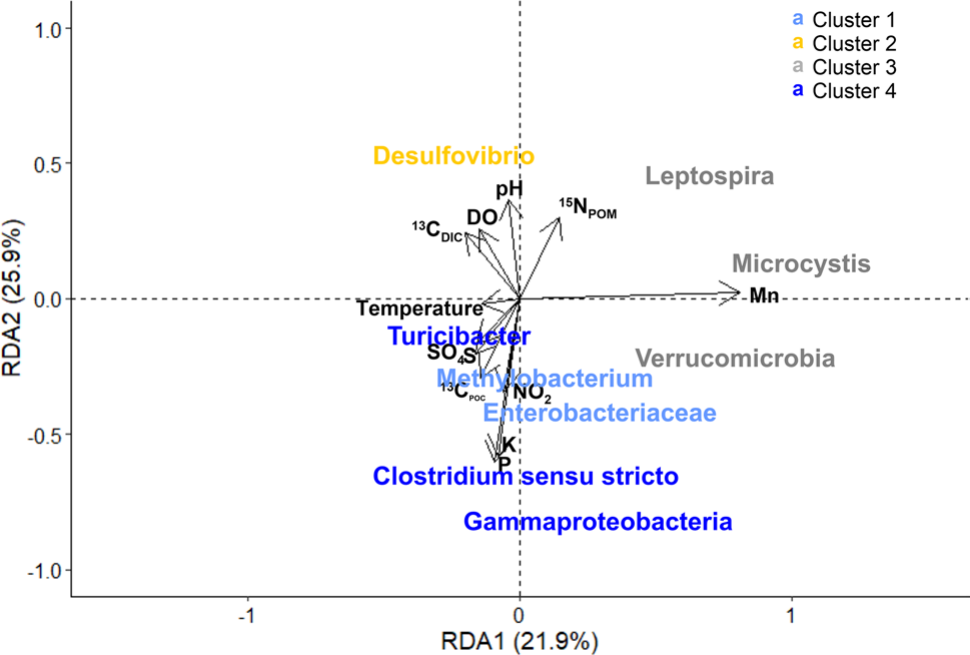
Redundancy analysis showing CKD related bacteria and water chemistry parameters. Microbial clusters assigned per Supplementary Figure 4.

The redundancy analysis in Fig. 4 shows the associations between CKD-related microbes present in this study which have been previously identified in higher concentrations in fecal samples from CKD patients, and water chemistry parameters. The four clusters represent microbes most closely associated with each other (Supplementary Figure 4).

RDA results (Fig. 4 and Supplementary Table 1) show that microbes associated with Cluster 1, 2 and 4 correspond with RDA2 which is most highly represented by phosphorus (P), potassium (K), pH and ^15^N of particulate organic matter (^15^N_POM_). Microbes associated with Cluster 3 correspond with RDA1 (*Microcystis*) and a combination of RDA1 and RDA2 (*Leptospira* and *Verrucomicrobia*). RDA1 is most highly associated with chemical parameters Mn and ^13^C_DIC_. The chemistry parameters used in the RDA explain approximately 47.8% of the data variability on RDA1 (x-axis) and RDA2 (y-axis) combined, suggesting that the variability of microbial abundances presented may also be highly influenced by other factors such as the abundances or presence of other OTUs not included in the analysis.

Linear regression analysis shows that *Microcystis*, *Turicibacter* and *Leptospira* are significantly positively correlated with pH (*p* = 0.03, *p* = 0.009 and *p* = 0.045 respectively). Cyanobacteria (including *Microcystis*) and *Leptospira* are positively correlated with DO (*p* = 0.018 and *p* = 0.009 respectively) and ^13^C_DIC_ (*p* = 0.023 and *p* = 0.017 respectively), and negatively correlated with ^13^C_POC_ (*p* = 0.025 and *p* = 0.003 respectively). We also observe a positive correlation between pH and ^13^C_DIC_ (*p* < 0.001) with increasing pH associated with increased ^13^C_DIC_ enrichment. *Methylobacterium* appear to be temperature dependent with increasing temperatures resulting in increasing *Methylobacterium* counts (*p* = 0.004). Nutrients K and P appear to be linked to Actinobacteria and Gammaproteobacteria (Fig. 4) however linear regression for P or K and Actinobacteria shows that the relationships are not significant (*p* > 0.05). In contrast, Gammaproteobacteria is significantly positively linked to increasing K and P (*p* = 0.007 and *p* < 0.001 respectively). It is noted that P concentrations are low for all samples (> 0.01 mg L^−1^–0.24 mg L^−1^).

## Discussion

Our water chemistry data confirm previous reports of increased levels of F in areas of high CKDu prevalence^16^, with 30% of household wells in the Medawachchiya region exceeding WHO recommendations, and more than half exceeding the Sri Lankan standard for potable water. In our association analysis of water chemistry and microbial abundances we find indication for a complex interaction between the microbial composition and water chemistry. The significant positive relationships observed between *Microcystis* and Cyanobacteria with DO and ^13^C_DIC_, as well as the negative correlation with ^13^C_POC_ suggests that these photosynthetic bacteria effect a significant increase in DO levels and play a role in carbon turnover due to their preferential fixation of ^12^C in CO_2_ via the Calvin cycle and subsequent release of oxygen. The utilization of the ^12^C-enriched carbon from the DIC pool results in the enrichment of ^13^C in the remaining DIC (i.e. more positive δ^13^C_DIC_ values). At the same time this causes an enrichment in of ^12^C in the POC pool (i.e. more negative δ^13^C_POC_ values) when algae utilize this DIC^40^. For δ^13^C_POC,_ we observe values as low as − 43.1‰, whilst δ^13^C_POC_ values observed from most aquatic environments (oceans, rivers, lakes) typically range from approximately − 30 to − 19‰^41–44^. Some plankton sources, however, have shown δ^13^C_POC_ values as low as − 42‰^45^. Fractionation of stable carbon isotopes of CO_2_ by Cyanobacteria has previously been observed in a lake system^46^. The enrichment of ^13^C_DIC_ caused by these photosynthetic bacteria is associated with a significant increase in pH, likely due to the reduction of carbonic acid resulting from the removal of dissolved CO_2_. The increase in Cyanobacteria and pH alters microbial composition resulting in an increase in *Turicibacter*, *Microcystis* and *Leptospira*. Significant correlations between DO, δ^13^C_DIC_ and δ^13^C_POC_ with *Letospira*, a non-photosynthetic bacterium is also observed, which appears to be due to its co-occurrence with Cyanobacteria (*p* = 0.022). The presence of *Microcystis* is relevant for domestic well waters being used for drinking water and where these waters are being used on crops. Cyanotoxins have been shown to bioaccumulate in plants which may ultimately impact on human health when consumed^47^. We suggest that further research focus on identifying the presence and concentrations of cyanotoxins in household wells. Major cyanotoxin groups to consider include cylindrospermopsin, nodularin, microcystins, anatoxins and saitoxins^48^. Recent research identified a negative association between the use of boiled water and CKD probability^49^, however short-term boiling does not statistically decrease the concentrations of cyanotoxins^50^. Further, behavioural differences within the same household particularly under economic constraints would merit closer scrutiny. We note that whilst low DO concentrations may indicate decreased Cyanobacterial abundances, cyanotoxins may build up and remain in high concentrations in stagnant waters. Furthermore, the presence of *Leptospira* in these waters indicates that well waters in the region may not only be harmful to drink, but also when used for bathing as this can allow *Leptospira* to enter the body through cuts or abrasions on the skin^51^.

Predicated on the notion that the microbiome of the entire alimentary system reflects host diet^52–54^ environment and lifestyle factors^55^ and is also strongly influenced by the quality and microbial content of drinking water^30,31,56–60^, we undertook a speculative interrogation of the microbial composition and abundance in the water contained in household wells based on bacterial phyla and genera that have previously found the gut microbiome of patients with chronic kidney disease. Bacterial abundance studies of the stool from patients with CKD show significantly reduced abundances of the phyla Actinobacteria, the abundances of Verrucomicrobia and Enterobacteriaceae family are higher. On the genus level, stool from CKD patients contains fewer *Akkermansia, Alloprevotella* and *Parasutterella*, and significantly higher *Enterococcus, Clostridium IV*, *Paraprevotella*, *Clostridium *sensu stricto and *Desulfovibrio*^37–39^. Higher abundances of potentially harmful Gammaproteobacteria, and lower abundances of beneficial genera including Rumincoccacea, *Bifidobacteria* and *Lactobacilli* have also been identified in CKD patients^38,39^.

While we found that most bacteria linked to the CKD OTUs of interest showed no clear relationship with P or K concentration in water, we observed a significant positive correlation between Gammaproteobacteria with increased K and P. One possible source of increased P could be the increased use of P fertilizer over the past few decades due to the intensification of crop production^61^. Research has demonstrated that soils treated with P fertilizers appear to have higher levels of Gammaproteobacteria^62^ which may leach into surface waters and shallow groundwaters.

*Methylobacterium* are opportunistic pathogens which can result in infection in immunocompromised patients including those with acute and chronic renal failure^63^. *Methylobacterium* infection in patients with kidney disease can manifest as peritonitis, pneumonia, abdominal pain and cloudy dialysate^64^. The optimum growth of these bacteria occurs between 25 and 30 °C with limited growth beyond 40°C^65^. We observe significant increases in this genus with increased temperature in our dataset (range = 27–32 °C). This may suggest that future climate change has the potential to alter spatial or temporal distributions of these pathogens. For example, presence of *Methylobacterium* in surface waters may decline in the warmer months with increasing ambient temperatures reaching closer to 40 °C, and increase in cooler months, or in cooler regions such as the south-central intermediate rainfall zone where average temperatures may be within the optimum growth range for Methylobacterium. The variable presence of Methylobacterium would then be similar to *Microcystis* and other pathogenic bacteria in these stagnant waters, in that drought zones can lead to the periodic build-up of extremely high levels of toxins or pathogens due to reduced flushing by rainfall and thus render the water of many household wells non-potable. This is particularly of concern considering the number of overall consecutive dry and wet days in Sri Lanka has increased and decreased respectively, with an expansion of the dry zone (average annual precipitation < 1750 mm) observed for the period 1961–1990 compared to 1911–1940^66^. In inland regions, research suggests rainfall during the wet seasons has declined by 40% from 1869 to 1993^66^, leading to increase stagnation of well waters in the central regions. In contrast, the wetter western regions of Sri Lanka are projected to experience increased rainfall during the Southwest Monsoon due to climate change. This is likely to result in a flushing of well waters in areas that are not currently affected by CKDu (Fig. 1).

## Limitations and conclusions

Traditional nosography (the classification and description of diseases) proceeds by establishing clinico-pathological correlations between clinical illness presentation, the exposure to one or more pathogen and the presence of histopathological or other biological features. In the case of CKDu/CINAC, this approach is limited by the delayed onset of the disease which forces the observer to back-extrapolate to past pathogen exposure. This approach cannot entirely exclude overlap with the clinical presentation of CKD, and the shared risk factor of low socio-economic status in these regions. It is not fully resolved whether the prevalence of farmworkers amongst the patients is inherent to their low socio-economic status and associated range of other unfavorable health determinants (nutritional and associated developmental deficits), or whether it is an independent risk factor associated with farm work-related pathogen exposure. The latter is supported by the observation in CINAC patients of variable lysosomal anomalies that are consistent with a toxic insult^67^. A formal link between one or more specific toxins to the lysosomal injury has not yet been established, as these or similar lysosomal anomalies tend to be observable across a range of disease conditions. Given the above, CKDu is now considered a epidemiological problem which is best captured by a pragmatic multilevel clinical case definition^1,68^.

A complementary approach to the isolation of single or multiple causes of disease, is to view health status as a complex emergent state resulting from adaptive social and biological network interactions^68,69^. In our analysis of drinking water, which represents a potentially important source of pathogenic exposure, we have therefore described the complete internal microbial ecology of household wells using operational taxonomic units derived from the internal gut microbial ecology of patients with CKD. While we do not have the data to comment on the extent to which the microbial signature of a given household well water might have determined that of the gut microbiome of the consumer, it is a non-trivial observation that there are indeed relevant bacteria in both microbial ecosystems.

This allows the speculation that the microbiome of a household well co-determines the gut microbiome of the user and thus could confer disease risk beyond that associated with the known pathogenic microbes that were also found in our samples. It is noted that the definition of the OTUs was limited by the available knowledge in regard to their presence in kidney disease and that a sequence-based taxonomy alone does not fully capture both genomic or functional-metabolic differences between bacteria^70^.

The outcomes of this study have led to many more questions around CKDu research. Further work should focus on identifying what the gut microbiome composition is in patients with CKDu compared to those with CKD. Investigations into the gut microbiome of healthy household members who consume the same water source would also provide much needed information. Furthermore, studies designed to understand intake volumes of water for consumers, bacterial densities, the presence and concentrations of cyanotoxins, how the well water microbiome changes seasonally and identifying the range of uses for the water from the household wells are needed. It would also be useful to compare samples from household wells in non-CKDu prevalent areas to the results from this study. Understanding the differences both in the quality of the water and the consumption patterns within individual households^71^ will also provide further information on the complex interactions between water and gut microbiomes.

It is likely that anticipated increases in water temperatures due to climate change, and increasing nutrients due to current farming practices, will impact on the quality of drinking water and, more speculatively, might change human gut microbial composition in its wake.

## Supplementary information

Supplementary Information.

## Data Availability

All data and code are available from the corresponding author upon request.
