# Supplementary material for: The water chemistry and microbiome of household wells in Medawachchiya, Sri Lanka, an area with high prevalence of chronic kidney disease of unknown origin (CKDu)
Source: Sci Rep. 2020 Oct 26;10:18295. doi: 10.1038/s41598-020-75336-7 (PMC7589467; doi:10.1038/s41598-020-75336-7)
Supplement: Supplementary file 1 — Supplementary Information. [file 41598_2020_75336_MOESM1_ESM.docx]

**Supplementary information for**

**The water chemistry and microbiome of household wells**

**in Medawachchiya, Sri Lanka, an area with high prevalence of**

**chronic kidney disease of unknown origin (CKDu)**

Authors: Liza K. McDonough^1,2,*^, Karina T. Meredith^1^, Chandima Nikagolla^3^, Ryan J. Middleton^1^, Jian K. Tan^1,4,5^, Asanga V. Ranasinghe^6^, Frederic Sierro^1,5^, Richard B. Banati^1,5^.

^1^ Australian Nuclear Science and Technology Organisation (ANSTO), New Illawarra Rd, Lucas Heights, NSW, 2234, Australia.

^2^ School of Biological, Earth and Environmental Sciences, UNSW Sydney, NSW, 2052, Australia

^3^ School of Civil and Environmental Engineering, Science and Engineering Faculty, Queensland University of Technology, Brisbane, QLD 4000, Australia

^4^ Charles Perkins Centre, University of Sydney, Sydney, NSW, Australia

^5^ Faculty of Medicine and Health, University of Sydney, Sydney, NSW, Australia

^6^ National Renal Disease Prevention and Research Unit, Ministry of Health, Colombo 10, Sri Lanka

*Corresponding author: lizam@ansto.gov.au


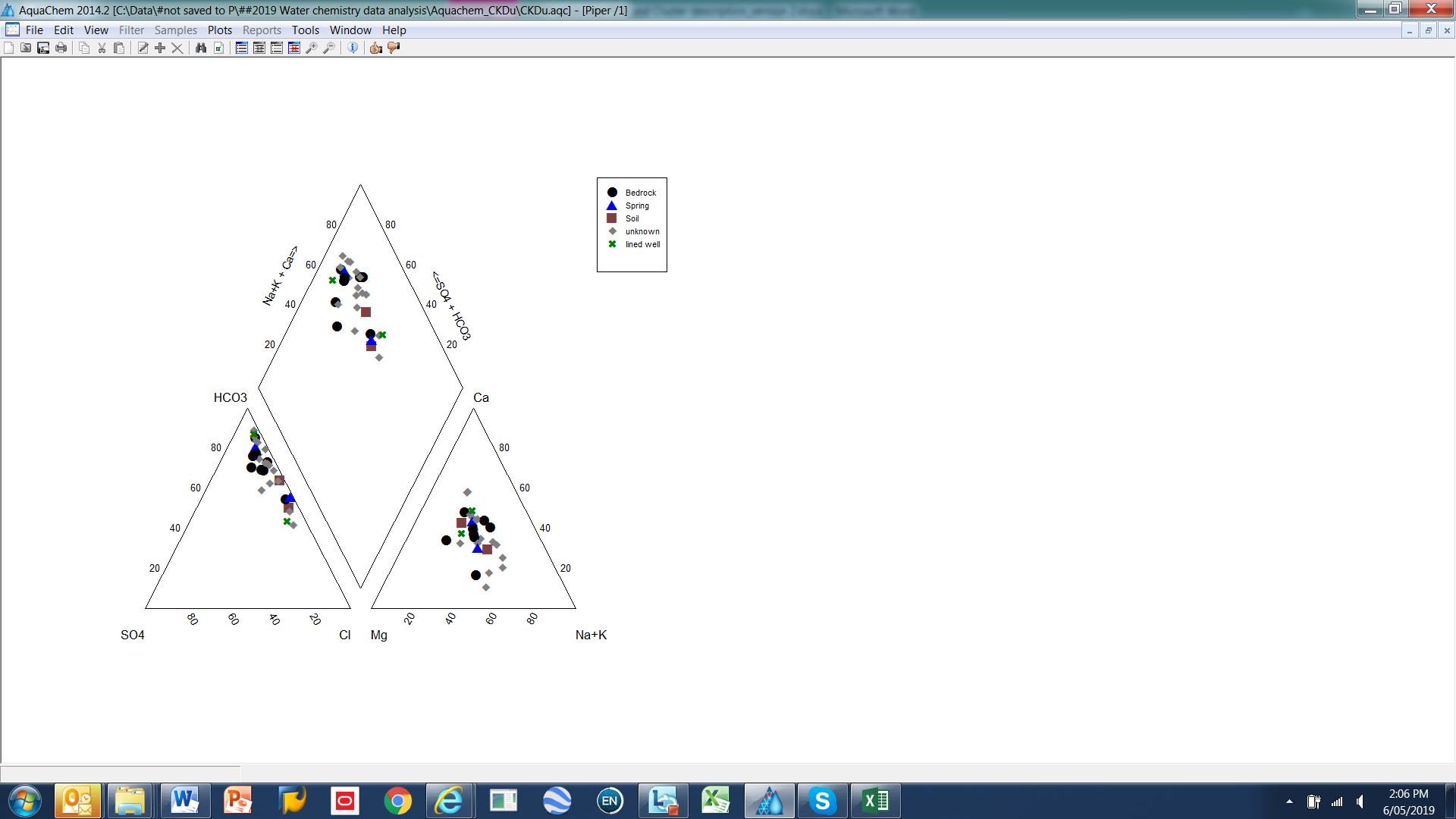


Supplementary Figure 1. Piper plot showing that the water types in the sampling area are calcium chloride and mixed water types ^1^.


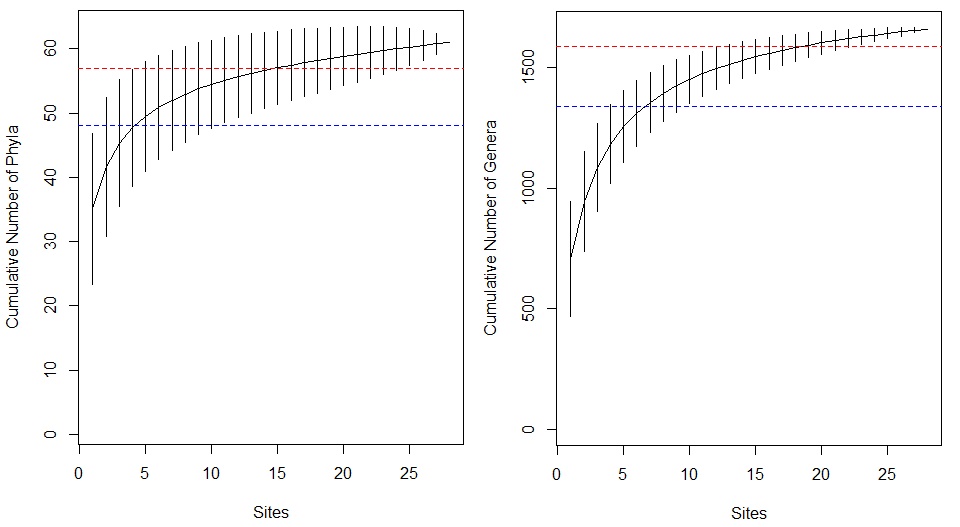


Supplementary Figure 2. Species accumulation curve showing the cumulative number of phyla (left) and genera (right) on the y-axis and the number of sites on the x-axis. Vertical lines represent error bars (standard error of the estimate). The blue and red dashed lines indicate 80% and 95% values respectively.


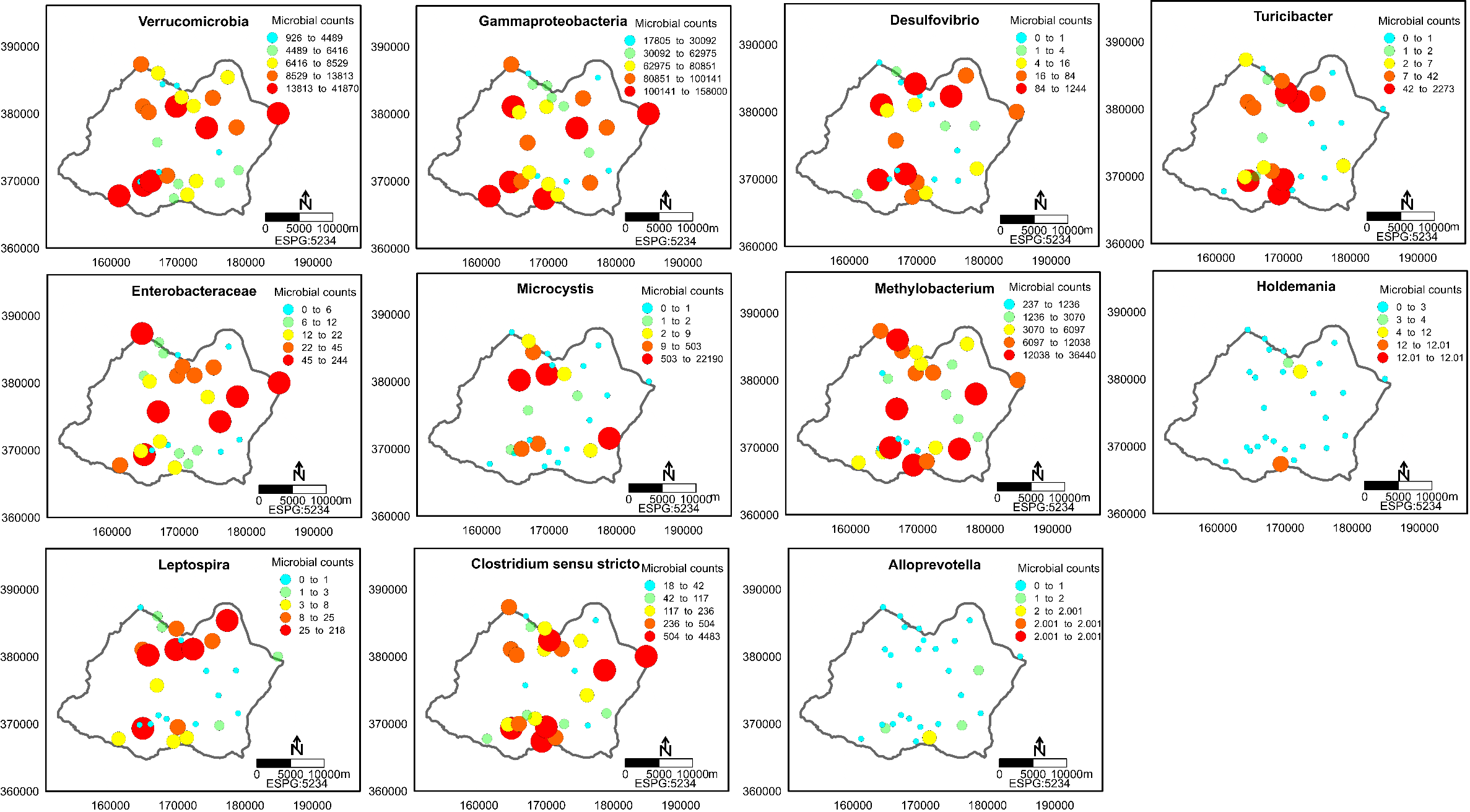


Supplementary Figure 3. Distribution maps of microbes associated with increased abundances in CKD patients. Figure prepared in Surfer v.11.0.642 (www.goldensoftware.com/products/surfer).


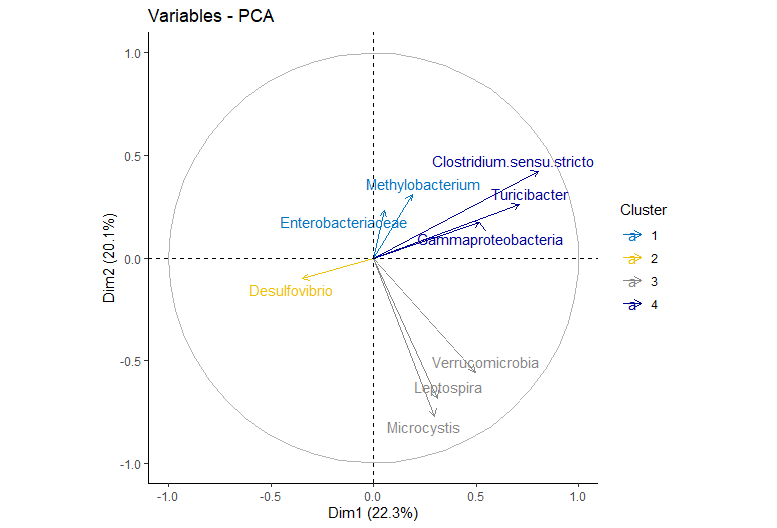


Supplementary Figure 4. PCA results showing microbial groupings of OTUs associated with increased abundance in CKD patients (Table 1). Groupings are based on k-means. OTUs present in less than 5 of 28 samples (*Alloprevotella*, *Holdemania*, *Clostridium IV* and *Enterococcus*) have been excluded.

Supplementary Table 1. Redundancy analysis (RDA) results. RDA1 shows high *Desulfovibrio*, ^13^C_DIC_, *Turicibacter*, S, SO_4_, DO, *Clostridium sensu stricto*, temperature, phosphorus and potassium, and low *Micocystis*, Mn, Verrucomicrobia, *Leptospira*, Enterobacteriaceae, Gammaproteobacteria, ^15^N_POM_ and *Methylobacterium*. RDA2 results show high Gammaproteobacteria, *Clostridium sensu stricto*, P, K, Enterobacteriaceae, NO_2_, ^13^C_POC_, Verrucomicrrobia, S, *Methylobacterium* and low *Desulfovibrio*, *Leptospira*, pH, ^15^N_POM_, DO, ^13^C_DIC_, *Microcystis* and Mn.

| Parameter | RDA 1 | RDA2 |
| --- | --- | --- |
| **OTUs** | | |
| Verrucomicrobia | -0.62345034 | 0.2643869 |
| Gammaproteobacteria | -0.15507029 | 0.8608205 |
| Enterobacteriaceae | -0.32322197 | 0.3424419 |
| Microcystis | -1.09110595 | -0.195664 |
| Leptospira | -0.59274978 | -0.4108641 |
| Clostridium sensu stricto | 0.14945663 | 0.6699367 |
| Desulfovibrio | 0.35867044 | -0.4845197 |
| Turicibacter | 0.1714794 | 0.1859118 |
| Methylobacterium | -0.03208607 | 0.1947559 |
| **Water Chemistry** | | |
| Temperature | 0.13851094 | 0.0192706 |
| pH | 0.04066324 | -0.36514822 |
| DO | 0.1506217 | -0.25605016 |
| ^13^C_DIC_ | 0.19989762 | -0.2435225 |
| ^15^N_POM_ | -0.14652156 | -0.29964295 |
| ^13^C_POC_ | 0.14456261 | 0.28966065 |
| K | 0.08227238 | 0.58133028 |
| Mn | -0.80859723 | -0.02249579 |
| p | 0.09415365 | 0.60395179 |
| S | 0.16246401 | 0.2043008 |
| NO_2_ | 0.05933255 | 0.34182746 |
| SO_4_ | 0.15712938 | 0.16323378 |

Supplementary References:

1 Piper, A. M. A graphic procedure in the geochemical interpretation of water-analyses. *Eos trans. AGU* **25**, 914-928, doi:10.1029/TR025i006p00914 (1944).
